# In Situ Polymer-Solution-Processed Graphene–PDMS Nanocomposites for Application in Intracranial Pressure Sensors

**DOI:** 10.3390/nano14050399

**Published:** 2024-02-21

**Authors:** Hua Hong, Junjie Zhang, Yuchen Zhu, Stephen D. Tse, Hongxuan Guo, Yilin Lai, Yubo Xi, Longbing He, Zhen Zhu, Kuibo Yin, Litao Sun

**Affiliations:** 1 SEU-FEI Nano-Pico Center, Key Lab of MEMS of Ministry of Education, Southeast University, Nanjing 210096, Chinaghx@seu.edu.cn (H.G.); helongbing@seu.edu.cn (L.H.); zhuzhen@seu.edu.cn (Z.Z.);; 2 Department of Mechanical and Aerospace Engineering, Rutgers University, Piscataway, NJ 08854, USA; sdytse@rutgers.edu

**Keywords:** graphene, polydimethylsiloxane, shear exfoliation, pressure sensor

## Abstract

Polydimethylsiloxane (PDMS) has emerged as a promising candidate for the dielectric layer in implantable sensors due to its exceptional biocompatibility, stability, and flexibility. This study introduces an innovative approach to produce graphene-reinforced PDMS (Gr-PDMS), where graphite powders are exfoliated into mono- and few-layer graphene sheets within the polymer solution, concurrently forming cross-linkages with PDMS. This method yields a uniformly distributed graphene within the polymer matrix with improved interfaces between graphene and PDMS, significantly reducing the percolation threshold of graphene dispersed in PDMS from 10% to 5%. As-synthesized Gr-PDMS exhibits improved mechanical and electrical properties, tested for potential use in capacitive pressure sensors. The results demonstrate an impressive pressure sensitivity up to 0.0273 kpa^−1^, 45 times higher than that of pristine PDMS and 2.5 times higher than the reported literature value. The Gr-PDMS showcases excellent pressure sensing ability and stability, fulfilling the requirements for implantable intracranial pressure (ICP) sensors.

## 1. Introduction

Injured tissue tends to experience hemorrhaging and edema, resulting in auxetic volume. Such processes pose unique challenges for the injured brain, as the brain resides within the confines of the skull, where compartment syndrome has already developed [1]. Hence, the long-term monitoring of intracranial pressure (ICP), which is exerted by fluids within the skull and on the brain tissue, is taken as a means of predicting and preventing inordinate cerebral perfusion pressure (CPP), and the relationship between the two is in the following given formula: CPP = mean arterial pressure (MAP) − ICP [2]. Thus, the monitoring of ICP plays a critical role in assessing brain damage and providing guidance on medication utilization in neurointensive care.

Several approaches have been implemented for ICP monitoring according to recent reviews [2,3]. The gold standard to measure intracranial compliance comprises catheter insertion into either of the ventricles and an external device to gauge strain, also known as the external ventricular drain (EVD) technique. Other effective measuring modalities include fiberoptic devices, pneumatic sensor devices, and strain gauge devices, among which a progressive method is a multifunctional wireless silicon sensor composed of bioresorbable materials that could support continuous monitoring for up to three days [4]. However, the aforementioned methods are rather cumbersome and could provoke complications and second injuries such as hemorrhaging and infection. More importantly, a paradox remains, as the need for longer monitoring maintenance within the ventricles arouses higher risks of complications, raising significant doubts about the necessity and overall impact of long-term monitoring [5]. Therefore, the ideal implantation site for long-term monitoring is the epidural space. Here, the sensor can be positioned outside the internal environment, offering a new avenue for continuous, long-term monitoring of ICP while mitigating the risk of secondary injuries [6].

Polydimethylsiloxane (PDMS) has been utilized in a wide range of biomedical applications, e.g., microfluidic devices [7], the fabrication of biomodels in disease research [8], and medical implants [9], because of its superb biocompatibility, excellent thermal stability [10], and cost effectiveness [11]. PDMS is also adopted as the encapsulation material in the ICP monitoring industry to reduce the thickness of conventional ICP monitoring sensors to 500 μm  [12]. Therefore, PDMS is a promising candidate for fabricating flexible sensors positioned in the epidural space. However, PDMS’s electrical and mechanical properties constrain its usage as a pressure sensor. Recently, nanofillers such as Au nanoparticles, graphene, and graphene derivatives, including graphene oxide (GO), reduced graphene oxide (rGO), and carbon nanotubes (CNTs), have been incorporated into polymers, aiming at addressing the limitations associated with electrical and mechanical properties [13,14]. Au nanoparticles are rather costly, limiting their usage in mass production. GO, which can be fabricated easily, is heavily decorated with oxygen-containing functionalities (OFs), rendering the material nonconducting [15]. rGO calls for extra reduction operations on GO, and the large-scale reduction method, chemical reduction, involves hazardous toxic reagents [16]. CNTs, which possess extraordinary mechanical properties, are toxic due to their dimensions, surface modifications, and protein corona [17], restricting their biomedical applications. Among all nanofillers, graphene stands out owing to its prominent features, i.e., large specific surface area, high tensile modulus, extraordinary electrical properties, biocompatibility, and well-established mass production methods [18]. Additionally, studies on the interphase of polymer composites confirm that graphene incorporated with PDMS has optimized interfacial strength and is endowed with multifunctionalities [19]. The essential requisite for harnessing graphene to modulate the mechanical and electrical properties of polymers lies in the quality of graphene additives. Currently, liquid-phase exfoliation (LPE) [20] is considered one of the most promising strategies for providing high-quality two-dimensional graphene suitable for nanofillers [21]. Generally, LPE involves normal and shear forces, including sonication, spinning, mixing, and ball milling [22,23].

Various methods have been employed to process graphene-enhanced nanocomposites, including dispersing graphene nanofillers into polymer solutions [24], blending melting polymers with graphene nanofillers [25], and layer-by-layer assembly [26]. Based on the characteristics of graphene and PDMS, solution mixing is the most suitable method for fabricating graphene-reinforced PDMS (Gr-PDMS). However, the major drawbacks associated with solution mixing include aggregation of graphene nanosheets [27] and the low permissible graphene concentration (~0.01 mg/mL) [28], constraining the properties of the composite matrix. A seminal work in this area utilizes a concentric-cylinder shearing device with in situ rheology to assess the extent of exfoliation to fabricate graphene-reinforced polymers, which achieves the unadulterated adhesion of the graphene nanoflakes in the polymer matrix without massive aggregation [29], providing insightful instructions for the preparation of Gr-PDMS.

Herein, an in situ processing approach based on liquid-phase shear exfoliation is employed to create Gr-PDMS composites suitable for use as a pressure-sensing layer in the flexible ICP monitoring sensors implanted in the epidural space. Through the in situ shear exfoliation of graphite powder in the polymer solution, as-exfoliated graphene flakes with intrinsic defects and dangling bonds can simultaneously form cross-bonding connections with the polymers’ backbones and branches, achieving this without contamination or substantial oxidation. The interfacial bonding within the composite matrix is thus strengthened, resulting in notable enhancements in mechanical and electrical properties while reducing the processing cost, time, and quantity of additives required. The samples produced in this manner are subjected to characterization using scanning electron microscopy (SEM), Raman spectroscopy, and X-ray photoelectron spectroscopy (XPS) to analyze their morphology and chemical structure. The electrical and mechanical properties of the as-synthesized Gr-PDMS are measured to demonstrate its capability to be used in a capacitive pressure sensor.

## 2. Materials and Methods

**Figure 1:**
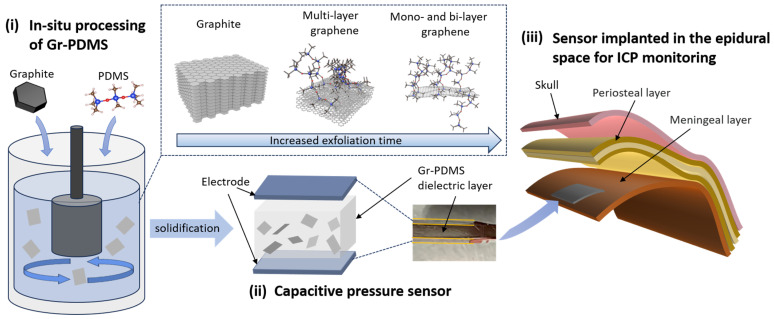
The preparation steps of Gr-PDMS-based capacitive pressure sensor and its potential application in ICP monitoring: (**i**) Gr-PDMS produced by the in situ processing method involving shear exfoliating graphite into mono- and few-layer graphene sheets, simultaneously forming cross-linkages with PDMS through edges and defects, (**ii**) capacitive pressure sensor fabricated by attaching parallel electrodes to a molded Gr-PDMS dielectric layer, and (**iii**) schematic of the implantation site of the pressure sensor for use in ICP monitoring.

The experimental steps for preparing Gr-PDMS using the in situ processing method are illustrated in Figure 1. The experiment utilizes dichloromethane (DCM) to dissolve PDMS powders, forming a viscous polymer solution for shearing. Since the PDMS/DCM solution exhibits a very high viscosity, the abovementioned concentric-cylinder shearing device cannot exfoliate and disperse graphene in such a polymer solution effectively. A high-shear mixer with a generator comprising a rotor and a stator is adapted to replace the inner cylinder. The high-speed blades of the rotor can create high shear force and high-speed fluid jets emitting from drain holes of the stator [30]. High-quality graphite (99.9% concentration) with a particle size of 2.5 μm at various loading weights (the corresponding Gr/PDMS ratio shown in Table 1) are dispersed in the PDMS/DCM and experience shear exfoliation, collisions, and jet cavitations generated by the high-shear mixer [30,31]. Expanding the scale of such a configuration poses no obstacles to the efficient production of Gr-PDMS composites. Exfoliated graphene nanoflakes form a uniform suspension in the polymer solution. Consequently, crosslinks are formed between the graphene sheets and polymer matrix within the solution through defect sites and dangling bonds of graphene.

The aforementioned setup comprises an inner high-shear generator and an outer cylinder, which are spaced closely (Figure 1i). The inner high-shear generator has a diameter of 28 mm with blades rotating at 3300 rpm for 1 h. The suspension is sealed within the system to prevent DCM evaporation. After high-shear mixing, the curing agent is mixed into the suspension in a 1:10 proportion with the host. After the suspension is stirred at 60 °C for 1 h, it is injected into molds and solidified at 60 °C for 3 h to produce Gr-PDMS composites.

The morphology of the Gr-PDMS samples is then investigated by ZEISS Sigma 300 SEM (Zeiss, Oberkochen, Germany). Raman spectra are obtained with a Raman Horiba LabRAM HR Evolution (Horiba, Irvine, CA, USA) using a 514 nm laser as the excitation source. XPS spectra are taken with a Thermo Fisher K-Alpha XPS (Thermo Fisher, Waltham, MA, USA) to analyze the chemical structures of the nanocomposites.

The mechanical properties of as-synthesized composites are measured using a setup with a Force Gauge (MARK-10 Series 5, Copiague, NY, USA). During the tensile test, to ensure that fracturing of the material occurs at the gauge (width = 12.5 mm, thickness = 1−3 mm), the specimens are prepared using dumbbell-shaped molds. A preload force of 150 N is applied to the specimens before testing to eliminate the structural instability resulting from the solidification process of materials. By setting an appropriate moving speed (0.5 mm/min), the specimens are uniformly stretched steadily. The initial length is measured between the two grips of the force gauge. The tensile force and displacement data are recorded. The tensile strength, elongation at break, and elastic modulus can be derived from the measured parameters using the corresponding formulas [13].

Samples of different Gr/PDMS ratios with an area of 12.5 mm × 50 mm and a thickness of 2 mm are assembled into parallel-plate capacitors by attaching copper electrodes to the top and bottom sides. The capacitance and resistance of the nanocomposites are measured using a precision LCR meter (Tonghui TH2826A, Changzhou, China) with a frequency range set to 10 kHz–1 MHz [32]. Samples are tested thirty times each to improve the accuracy. Then, the dielectric constant and conductivity of Gr-PDMS can be derived from the corresponding formulas [32].

Afterward, the pressure sensing capability of Gr-PDMS nanocomposite is demonstrated by measuring the varying capacitance of as-fabricated parallel-plate capacitors when external forces are applied and removed repeatedly. The pressure sensitivity, restorability, and repeatability are the major features of the Gr-PDMS-based pressure sensor device. Moreover, the elastic modulus and permittivity of Gr-PDMS fabricated under various rotation speeds (i.e., 2200 rpm, 3300 rpm, 4400 rpm) and mixing times (i.e., 30 min, 60 min, 90 min) are captured to analyze the effects of processing conditions on the properties of the nanocomposites.

## 3. Results

### 3.1. Characterization

**Figure 2:**
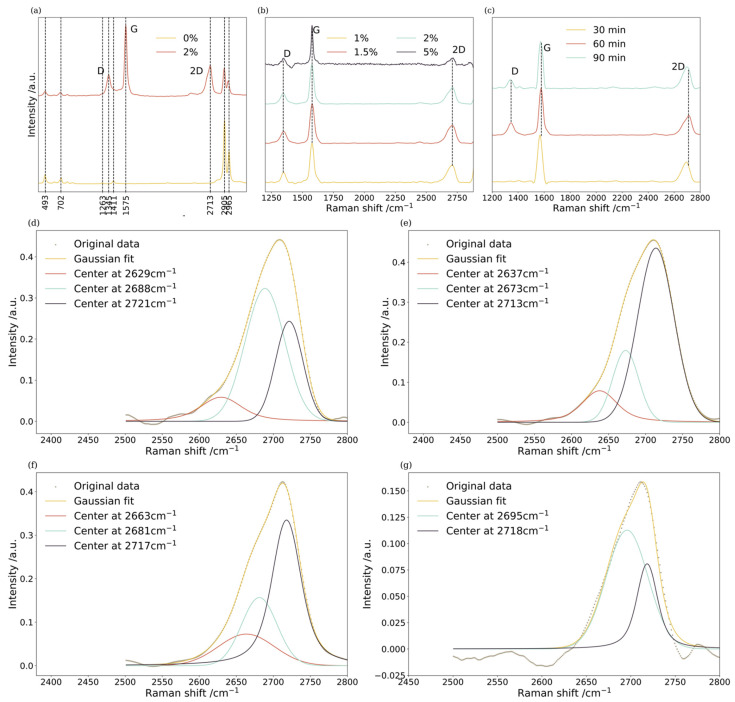
Raman spectra of (**a**) Gr-PDMS (Gr/PDMS = 2%) and pristine PDMS (Gr/PDMS = 0%), (**b**) composites with Gr/PDMS ratios of 1%, 1.5%, 2%, and 5%, (**c**) Gr-PDMS (Gr/PDMS = 2%) with different duration of in situ processing. Gaussian curve fitting for Raman 2D peaks of composites with Gr/PDMS ratios of (**d**) 1%, (**e**) 1.5%, (**f**) 2%, and (**g**) 5%.

The Raman spectra of Gr-PDMS with various concentration ratios elucidate the effect of the amount of graphite additives on the structures of the composites (Figure 2). As noted in the spectra, the following PDMS characteristic peaks could be observed: Si–O–Si at $493\;cm^{-1}$, Si–C stretch at $702\;cm^{-1}$, –CH_3_ symmetry stretch at $2905\;cm^{-1}$ and –CH_3_ asymmetry stretch at $2965\;cm^{-1}$ [33]. Meanwhile, the graphene characteristic peaks appear in the following wavebands: a G peak at around $1580\;cm^{-1}$, a D peak near $1350\;cm^{-1}$, and a 2D peak near $2700\;cm^{-1}$. Typically, the $I_{D}/I_{G}$ intensity ratios indicate disorders within the graphene layers, while the intensity ratios of $I_{2D}/I_{G}$ and the positions of the G and 2D peaks correlate to the number of graphene layers [34,35].

The $I_{2D}/I_{G}$ intensity ratio sits between 0.41 and 0.49 when the Gr/PDMS ratio is relatively low (1–2%), confirming exfoliated graphene with less than ten layers [36]. The $I_{2D}/I_{G}$ ratio drops notably when the Gr/PDMS ratio reaches 5%, indicating that more graphite additives might increase the number of layers of exfoliated graphene under the same experimental conditions. Simultaneously, the $I_{D}/I_{G}$ intensity ratio is found to be confined to 0.25–0.3 when the Gr/PDMS ratio stays between 1% and 2%. In comparison, the $I_{D}/I_{G}$ ratio drops to 0.16 as the Gr/PDMS ratio reaches 5%, indicating fewer defects are created within the exfoliated graphene as the concentration ratio rises. However, the defect sites within graphene sheets play vital roles in forming cross-bonding connections between graphene and polymers. Extended shearing duration can also contribute to the rise in $I_{D}/I_{G}$ and $I_{2D}/I_{G}$ ratio (Figure 2c).

Apart from estimating the number of graphene layers based on the $I_{2D}/I_{G}$ ratio, fitting the Raman 2D peak with Gaussian–Lorentzian curves is also an effective way to assess the number of graphene layers [37]. It is found that a monolayer and bilayer are located at peak ~2647 $cm^{-1}$, few layer (3–5 layers) at peak ~2690 $cm^{-1}$ and a multilayer at peak ~2727 $cm^{-1}$. Accordingly, 2D peaks of as-synthesized Gr-PDMS are fitted with Gaussian–Lorentzian curves, as shown in Figure 2e–g. At a low Gr/PDMS ratio of 1.5%, peaks near 2640 $cm^{-1}$ are observed to be dominant, corroborating the abundance of monolayer and bilayer graphene sheets. In contrast, when the Gr/PDMS ratio is further elevated, peaks near 2640 $cm^{-1}$ are replaced by peaks near 2690 $cm^{-1}$, indicating few-layer and multilayer graphene. With increased graphite loading, much more time may be needed to exfoliate to the same degree as for lower loading.

**Figure 3:**
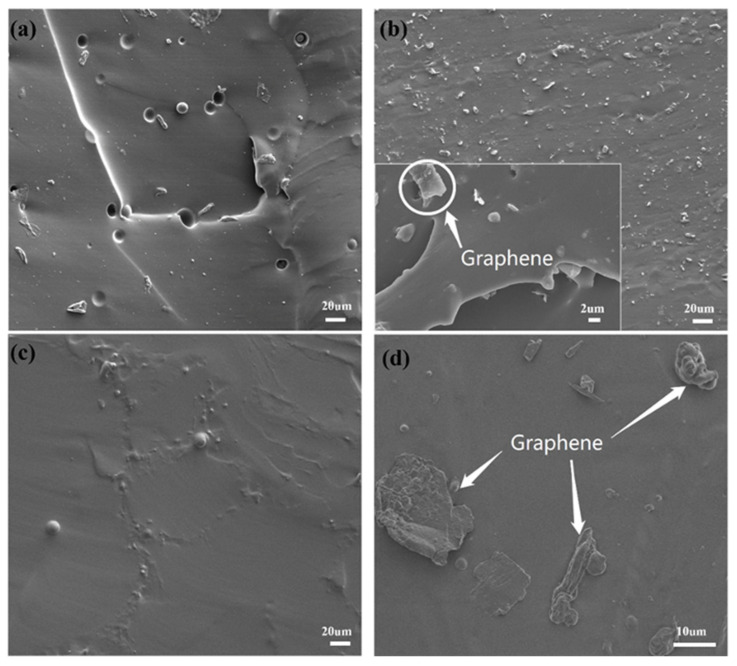
SEM images of the cross-section areas of samples with Gr/PDMS ratios of (**a**) 0%, (**b**) 2%, (**c**) 5%, and (**d**) 10%.

The SEM images shown in Figure 3 divulge the morphologies of Gr-PDMS nanocomposites. The interface between graphene flakes and PDMS is visible in the cross-section view. The pristine PDMS exhibits grain boundaries and pores with a diameter between 10 and 20 μm under a low-magnitude SEM image (Figure 3a). Such a morphology is attributed to the temperature variation during the curing process. When the Gr/PDMS ratio increases to 2%, the pore structures disappear, but some graphene flakes attached to the edges of the discontinuous island-like structures can be seen in high-resolution images (Figure 3b). As the Gr/PDMS ratio approaches 5%, the cross-section becomes smooth with no apparent island or pore structures (Figure 3c). Thus, such a result suggests that graphene nanoflakes are uniformly dispersed and interconnected within the PDMS matrix, forming a compact structure. The significant difference in electronegativity between Si and O atoms in the PDMS backbone results in the partial ionic character of the *Si-O* bonds. Strong interactions between the exposed bonds of dissolved PDMS subunits and graphene during exfoliation processing promote the dispersion of graphene in the PDMS and the formation of the composite material [38]. Graphene agglomerations ranging from 10 to 20 μm are observed when the Gr/PDMS ratio is further increased to 10% (Figure 3d), implying a decrease in graphene dispersibility owing to a higher nanofiller concentration.

**Figure 4:**
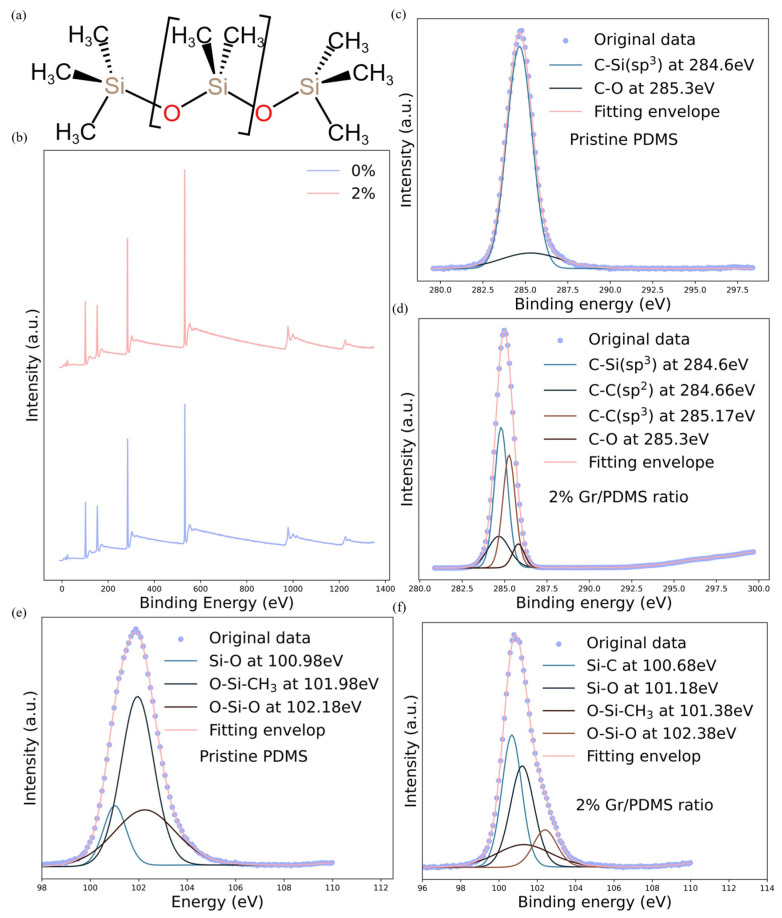
(**a**) The structural formula of PDMS. (**b**) XPS spectra of Gr-PDMS (Gr/PDMS = 2%) and pristine PDMS (Gr/PDMS = 0%). The representative peak fitting of *C1s* peaks of samples with Gr/PDMS ratios of (**c**) 0% and (**d**) 2% Gr/PDMS in high-resolution XPS spectra. The representative peak fitting of *Si2p* peaks of samples with Gr/PDMS ratios of (**e**) 0% and (**f**) 2% Gr/PDMS ratio.

The chemical structures of samples are further investigated by comparing the XPS spectra between the as-synthesized composite matrix dispersed with few-layer graphene (Gr/PDMS = 2%) and pristine PDMS. In the XPS spectra of both PDMS and Gr-PDMS, several peaks appear simultaneously, i.e., *O1s* at 532 eV, *C1s* at 284 eV, *Si2p* at 102 eV and *Si2s* at 152 eV (Figure 4). Under high-resolution XPS spectra, the *C1s* peak centering at 284.8 ev could be fitted into 4 components, i.e., $C–C\;\left(sp^{3}\right)$ (284.66 ev), $C–C\;\left(sp^{2}\right)$ (285.17 ev), C–O (285.62 ev) and C–Si (284.8 ev), and the *Si2p* peak centering at 102.8 eV could also be fitted into 4 components, i.e., $O–Si–CH_{3}$ (101.38 ev), Si–O (101.18 ev), O–Si–O (102.38 ev) and Si–C (100.67 ev) [39]. The detailed changes in the concentration of each chemical bond are calculated by dividing the corresponding peak area by the sensitivity factor [40], as listed in Table 2 and Table 3. The concentrations of $C–C\;\left(sp^{3}\right)$ and $C–C\;\left(sp^{2}\right)$ bonds increase significantly after adding graphite powders. Notably, the concentration of $C–C\left(sp^{3}\right)$ bonds exceeds twice the amount of $C–C\;\left(sp^{2}\right)$, while pristine graphene contains mostly $C–C\left(sp^{2}\right)$ bonds. Meanwhile, the concentration of C–Si bonds in the PDMS backbone also drops slightly, implying that some of the C–Si bonds break during the exfoliation process. Therefore, the significant increase in the concentration of $C–C\;\left(sp^{3}\right)$ may be due to the formation of interfacial carbon bonds between exfoliated graphene sheets and PDMS backbones. On the contrary, conventional mixing methods utilize graphene powders with inactive surfaces (from termination of dangling bonds during handling) instead, so the interfacial bonding between the PDMS backbone and graphene is weak, possibly leading to an ineffective interfacial stress transfer [41]. Additionally, the concentration of C–O drops to nearly half the initial amount, indicating that the in situ process does not lead to further oxidation of graphene, preserving its unique features. As displayed in Table 3, after introducing graphene, the concentrations of $O–Si–CH_{3}$ and O–Si–O bonds drop to less than half while those of Si–C and Si–O rise tremendously. Such changes further demonstrate that graphene reacts with PDMS branches to form linkages during the polymer solution process, as $O–Si–CH_{3}$ bonds break down, and the graphene carbon atom can react with the silicon atom to form a Si–C linkage. Under the influence of high shear forces in the polymer solution, the $–CH_{3}$ branches of PDMS easily break and simultaneously crosslink with graphene, resulting in better interfacial contact between graphene and PDMS than with the conventional solution mixing method. Thus, the in situ exfoliation processing plays a crucial role in enhancing the performance of the composite matrix.

### 3.2. Mechanical Properties

**Figure 5:**
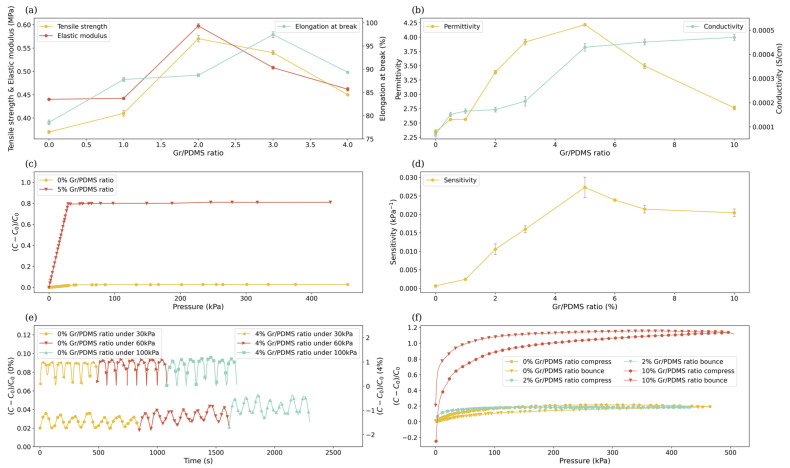
(**a**) Tensile strength, elastic modulus, elongation at break, (**b**) permittivity, and conductivity of Gr-PDMS with various Gr/PDMS ratios. (**c**) The capacitance changes of Gr-PDMS with 0% and 5% Gr/PDMS ratios under a pressure range of 0–430 kPa. (**d**) The sensitivity of Gr-PDMS at various Gr/PDMS ratios. (**e**) The capacitance changes of Gr-PDMS with 0% and 4% Gr/PDMS ratios during cyclic pressure loadings. (**f**) The recovery capability of Gr-PDMS with 0%, 2%, and 4% Gr/PDMS ratios.

Pristine PDMS has a relatively long bond length (1.64 Å) and a large *Si-O* bond angle ($130–160^{\circ }$), resulting in superb flexibility but also fragility. Through the dispersing of graphene, nanocomposites can obtain enhanced mechanical properties. The mechanical properties of nanocomposites with different Gr/PDMS ratios are measured via tensile tests. When the Gr/PDMS ratio increases, the overall mechanical properties of the nanocomposites improve at first, reaching a peak at 2% (Figure 5a). At the peak point, the tensile strength reaches 0.571 Mpa, which is 1.56 times higher than that of the pristine PDMS, and the elastic modulus reaches 0.597 Mpa, which is 1.36 times higher. The maximum elongation at break appears when the Gr/PDMS ratio is 3% because graphene nanofillers, functioning as mechanical enhancement, are dispersed into the structure of PDMS, creating strong chemical bonding when the nanofiller concentration is relatively low. Under such conditions, graphene layers and uniformity play critical roles in improving the mechanical properties. However, once the Gr/PDMS ratio surpasses a threshold (approximately between 2% and 3%), as the layer number of graphene increases, the stacking and agglomeration of graphene sheets escalate, leading to more crack propagation [42]. As a result, the tensile strength decreases to 0.445 MPa when the Gr/PDMS ratio is 5%.

Additionally, the rise of graphene–graphene interfaces constrains the motion of the polymer’s chemical chains, so the elongation at break decreases to 89.329% at a 5% Gr/PDMS ratio [43]. Meanwhile, major improvements in the elastic modulus are achieved not by alleviating the rotation speed but by extending the shearing time, indicating that shearing time contributes significantly to the uniform dispersion of graphene nanoflakes. As discussed above, graphene additives can improve the mechanical properties of the polymer composites, but the distribution and the layer number of exfoliated graphene determine the enhancement. Therefore, the shear exfoliation conditions of the liquid process need to be further investigated to achieve optimal properties.

### 3.3. Electrical Properties

The permittivity is mainly attributed to the polarization induced by the electric field. Typically, graphene-reinforced nanocomposites tend to be materials with a high potential dielectric constant, primarily because of the conductivity mismatch between the filler and the matrix. The conductivity mismatch could trigger various polarization mechanisms at the micro-level, e.g., electronic polarization, dipole polarization, and interfacial polarization, leading to the accumulation of electric charges [44]. Hence, the dielectric properties of nanocomposites are largely influenced by the nature of the interface between conductive fillers and the matrix, the surface area of the conductive fillers, and their inherent electrical conductivity. The permittivity decreases as the frequency increases within the high-frequency range, revealing the composites’ electrical conduction and dielectric relaxation behaviors [45]. The decrease is a consequence of some dipoles ceasing to reorientate under high frequency and losing their ability to hold electric charges. When the Gr/PDMS ratio is between 0 and 5%, the permittivity increases with the graphene concentration (Figure 5b), indicating that more electric charges accumulated at the graphene–PDMS interfaces cause stronger polarization. However, as the Gr/PDMS ratio exceeds 5%, the permittivity of the nanocomposite begins to decrease, implying the transition from a non-percolating state to a percolating state [46]. Under the percolating state, not only is graphene within the same cluster connected, but different graphene clusters are interconnected, resulting in significant current leakage and, thus, dielectric loss [47,48]. The composite reaches the percolating state at a 5% Gr/PDMS ratio, which is far below the current state-of-the-art processing technique (10%) [49], revealing that our method can significantly improve the dispersion of the exfoliated graphene. Moreover, enhancements in permittivity may be achieved by increasing the rotation speed duration.

In a polymer composite, if the concentration of conductive filler exceeds a certain threshold, the composite’s conductivity can’t be improved significantly with further addition of fillers. This threshold is called the percolation limit/threshold of the composite [50]. For as-synthesized Gr/PDMS composites, when the graphene content is in the range of 0~3 wt%, which is far below the threshold ~5 wt%, the conductive fillers are isolated from each other in the composite, forcing electrons to migrate through the “tunneling” phenomenon [44]. The conductivity of the nanocomposite is slightly increased along with the graphene content (Figure 5b). As the percolation threshold is approached, the conductive network is gradually formed through the added conductive fillers physically contacting one another in the composite. As a result, the transport of electrons changes from “tunneling” of electrons to electron conduction, leading to a significant jump in the conductivity. The result evinces the transition from a non-percolating state to a percolating state at a graphene content of ~5 wt% [51]. After the conductive network is completed at the percolation threshold, the conductivity only increases slightly with the further addition of graphene. Even though Gr-PDMS with a 10 wt% graphene content having a conductivity of $4.7\times 10^{-4}$ S/cm is much less than the excellent biocompatible conducting polymer PPy enhanced by graphene exhibiting a conductivity up to 7.93 S/cm, the negative permittivity limits the use of conductive polymers to bioelectrodes rather than dielectric layer of sensors [52,53].

### 3.4. Pressure Sensing Abilities of Gr-PDMS Nanocomposites

As graphene fillers are isolated by very thin polymer layers around the percolation threshold of Gr-PDMS, a large number of microcapacitor structures can be formed in the composite [54]. When external forces are applied, the average distance between fillers decreases, increasing the possibility of electron tunneling and the formation of microcapacitors at the interface [55]. The dispersed graphene effectively enhances the pressure sensitivity of nanocomposites in the range of 0–30 kPa load. The capacitance change rate shows a linear correlation with the applied load, making it an ideal dielectric for strain and pressure sensors [56]. However, when the load pressure surpasses 30 kPa, the large deformation results in a nonnegligible shearing force originating from the edge of the sensing material, breaking the linear relationship between pressure and capacitance change (Figure 5c) [57]. As shown in Figure 5d, the capacitance of nanocomposites with a particle load near 5% changes most rapidly in response to external forces. The as-produced nanocomposite with a 5% Gr/PDMS ratio exhibits the optimal pressure sensitivity, reaching $0.0273\;kpa^{-1}$, which is 45 times higher than pristine PDMS and 2.5 times higher than that in the literature (0.00936 $kPa^{-1}$) [58]. More importantly, such a level of sensitivity can induce a detectable change in capacitance under the necessary pressure accuracy in the ICP monitoring industry (266.645 Pa) [2]. Compared with the piezoresistive graphene-based BP/PVA used in a strain sensor, whose relative resistance change is limited to less than 1%, the relative capacitance change of as-synthesized Gr-PDMS can reach over 100%. Additionally, PVA is water-soluble and cannot be used in vivo for long-term monitoring [59,60].

The stability of the nanocomposites is studied by analyzing the capacitance change during cyclic pressure application. Gr-PDMS nanocomposites exhibit nearly identical waveforms to the pristine PDMS when the load is applied and removed repeatedly. Such a result demonstrates that the material possesses excellent stability and repeatability, which are essential properties for sensors (Figure 5e). The restorability of the nanocomposites is tested by measuring the deformation of the material once the external load is removed. Pristine PDMS exhibits significant changes in capacitance during the compression and rebound progression, revealing its non-restorability because of adhesion hysteresis, i.e., it takes time for polymer chain reorganization at the composite surface [61]. The adhesion hysteresis is largely reduced for Gr-PDMS nanocomposites, reaching the minimum at 2% particle load, namely the compression curve and rebound curve almost overlap (Figure 5f). However, the further increase in the graphene concentration results in the decline of the restorability, reaching the bottom at a 10% particle load, implying that crosslinks are created among dispersed graphene clusters, as discussed before.

## 4. Conclusions

A novel in situ processing method is presented for fabricating a Gr-PDMS polymer matrix composite, offering distinctive advantages in time, cost-efficiency, and scalability. The modified setup provides a dynamic liquid-phase exfoliation process involving shear force, jet cavitation, and collision, enhancing the breakdown of graphite, the creation of graphene defects, and the dispersion of graphene in the composite matrix. Characterizations of the Gr-PDMS composites reveal that graphite powders are exfoliated into mono- and few-layer graphene in a polymer solution, simultaneously creating unadulterated linkages with PDMS backbones and branches. Such an in situ process ensures a homogenous distribution of graphene within the polymer matrix. More importantly, the formation of interfacial carbon bonds between exfoliated graphene and PDMS backbones is scrutinized. The percolation threshold of Gr-PDMS is significantly halved to 5% (compared to that reported by others), and major improvements in mechanical and electrical properties are observed in the nanocomposites. Specifically, the tensile strength of as-synthesized Gr-PDMS reaches 0.571 MPa because of the formation of stronger chemical bonding than that produced via the conventional mixing method. The tensile strength and elastic modulus of such Gr-PDMS are increased by 54% and 36%, respectively, while that of the composite prepared by an in situ processing setup of concentric cylinders can only be increased by up to 28.6% and 31% [29]. Meanwhile, the processing time is significantly reduced to 1 h from the reported 2–6 h due to the improved effects of exfoliation. Additionally, a relative permittivity of 4.216 is achieved through the polarization at the interface of graphene nanofillers and polymer matrix. The pressure-sensing ability is tested by fabricating a sandwich-shaped capacitor with GR-PDMS as the dielectric layer. The prototype exhibits exceptional sensitivity, which is 45 times higher than pristine PDMS and 2.5 times higher than that in the literature. Such results demonstrate that the Gr-PDMS-based dielectric layer with excellent biocompatibility, stability, and flexibility, as well as pressure sensitivity, is promising for applications in ICP monitoring devices implanted in the epidural space. Further study will be conducted on implantable pressure sensors based on the Gr-PDMS dielectric layer. Our investigation of novel implantable pressure sensors based on flexible devices could inspire further research on functional ICP monitoring devices. Moreover, the in situ processing method demonstrated here poses great potential in the mass production of various polymer composites enhanced by two-dimensional nanofillers. Polylactic acid (PLA) has long been used in bioimplants and biomedical products due to its excellent biocompatibility and biodegradability. The in situ processing method is a promising approach to enhance the mechanical, electrical, and biomedical properties of PLA using graphene to achieve more applications in implantable bioelectronics and tissue engineering.

## Figures and Tables

**Table 1:** The graphite-to-PDMS weight ratios (Gr/PDMS) for the preparation of Gr-PDMS composites.

Gr/PDMS (%)	Graphite Particles (g)	PDMS (g)
0.0	0	30
0.5	0.15	30
1.0	0.3	30
1.5	0.45	30
2.0	0.6	30
3.0	0.9	30
4.0	1.2	30
5.0	1.5	30
6.0	1.8	30
7.0	2.1	30
10.0	3.0	30

**Table 2:** Concentrations of chemical bonds within the *C1s* peak.

Gr/PDMS (%)	$C–C\left(sp^{3}\right)$ (%)	$C–C\left(sp^{2}\right)$ (%)	*C–Si* (%)	*C–O* (%)
0.0	0	0	85.26	13.87
2.0	33.45	16.12	43.78	6.65

**Table 3:** Concentrations of chemical bonds within the *Si2p* peak.

Gr/PDMS (%)	*O–Si–CH_3_*	*O–Si–O*	*Si–C*	*Si–O*
0.0	53.73	32.87	0	13.40
2.0	17.16	12.20	36.81	33.83

## Data Availability

Data are contained within the article.
